# Comprehensive Analysis of *SRO* Gene Family in *Sesamum indicum* (L.) Reveals Its Association with Abiotic Stress Responses

**DOI:** 10.3390/ijms222313048

**Published:** 2021-12-02

**Authors:** Aili Liu, Mengyuan Wei, Yong Zhou, Donghua Li, Rong Zhou, Yanxin Zhang, Xiurong Zhang, Linhai Wang, Jun You

**Affiliations:** 1 Key Laboratory of Biology and Genetic Improvement of Oil Crops, Oil Crops Research Institute, Ministry of Agriculture and Rural Affairs, Chinese Academy of Agricultural Sciences, Wuhan 430062, China; liuailihappy@126.com (A.L.); weimengyuan@163.com (M.W.); ldh360681@163.com (D.L.); rongzzzzzz@126.com (R.Z.); zhangyanxin@caas.cn (Y.Z.); zhangxr@oilcrops.cn (X.Z.); 2 Key Laboratory of Crop Physiology, Ecology and Genetic Breeding, Ministry of Education, College of Bioscience and Bioengineering, Jiangxi Agricultural University, Nanchang 330045, China; yongzhou@jxau.edu.cn

**Keywords:** sesame, SIMILAR TO RCD-ONE (SRO), transgenic yeast, transcription factor, phylogenetic analysis, gene expression

## Abstract

SIMILAR TO RCD-ONEs (SROs) comprise a small plant-specific gene family which play important roles in regulating numerous growth and developmental processes and responses to environmental stresses. However, knowledge of SROs in sesame (*Sesamum indicum* L.) is limited. In this study, four *SRO* genes were identified in the sesame genome. Phylogenetic analysis showed that 64 SROs from 10 plant species were divided into two groups (Group I and II). Transcriptome data revealed different expression patterns of *SiSROs* over various tissues. Expression analysis showed that Group II *SROs*, especially *SiSRO2b*, exhibited a stronger response to various abiotic stresses and phytohormones than those in Group I, implying their crucial roles in response to environmental stimulus and hormone signals. In addition, the co-expression network and protein-protein interaction network indicated that SiSROs are associated with a wide range of stress responses. Moreover, transgenic yeast harboring *SiSRO2b* showed improved tolerance to salt, osmotic and oxidative stress, indicating *SiSRO2b* could confer multiple tolerances to transgenic yeast. Taken together, this study not only lays a foundation for further functional dissection of the *SiSRO* gene family, but also provides valuable gene candidates for genetic improvement of abiotic stress tolerance in sesame.

## 1. Introduction

Abiotic stresses are major factors limiting crop growth and productivity. Plants have evolved numerous families of regulators in their genomes that can modulate the expression of downstream genes, further regulate the signaling transduction networks and finally cope with adverse stress conditions [1,2]. The SIMILAR TO RCD-ONE (SRO) is a group of small plant-specific gene family that is widely involved in plant growth and development, as well as plant response to various environmental stimulants such as salt, drought, cold, UV-B irradiation, and heavy metals [3,4,5,6]. The structure of SRO family members is quite conserved. SRO proteins possess two characteristic features consisting of a conserved poly (ADP-ribose) polymerase (PARP) domain (PF00644) and a *C*-terminal RST (RCD-SRO-TAF4) domain (PF12174) [3]. In addition, some SRO proteins also have an additional *N*-terminal WWE domain (PF02825) [3]. Previous studies demonstrated that the RST domain is indispensable for the interaction between SRO proteins and different transcription factors [7,8]. Most SROs possess a PARP domain with an unusual catalytic triad motif and do not have PARP activity, except for wheat SRO protein Ta-sro1 [3,9].

The first characterized *SRO* gene was *Arabidopsis RADICAL-INDUCED CELL DEATH1* (*RCD1*). The *rcd1* mutant was found sensitive to O_3_ and showed extensive formation of lesions [10]. Meanwhile, Belles-Boix et al. [11] reported RCD1 (they named it as CEO1) could complement the oxidative stress sensitivity in yeast. Further reports revealed that loss-of-function mutation in *AtRCD1* lead to highly pleiotropic phenotypes including developmental defects (i.e., early flowering, aberrant leaf, root, and rosette morphology), changed plant stress resistance to various stresses (i.e., UV-B irradiation, salt, freezing, drought, oxidative stress), and altered responses to diverse hormones including jasmonic acid (JA), abscisic acid (ABA), and ethylene [5,7,10,12,13,14,15,16]. AtRCD1 was found to interact with SOS1 and several transcription factors, including DREB2A, thus protect plants against various stress conditions [7,14]. Subsequently, five other *Arabidopsis SRO* genes (*AtSRO1–5*) were identified and functionally characterized [3]. For example, as the closest homolog to *AtRCD1*, *AtSRO1* has part of the role of *AtRCD1* during development [15,16]. Compared to the *rcd1* mutant, *sro1* mutant showed normal development, whereas the double mutant of *AtRCD1* and *AtSRO1* displayed severe growth phenotypes. In addition, they play redundant and diverse roles in response to different stress conditions. Both of *rcd1-1* and *sro1-1* mutants showed resistance to osmotic stress, while they have opposite phenotypes to apoplastic ROS and salt stress [7,15]. SROs have also been isolated and functionally characterized in various plant species. For example, rice OsSRO1c plays a key role in drought and oxidative stress by regulating of stomatal closure and H_2_O_2_ level through interactions with different TFs [6,8]. Recently, the *OsSRO1c*/*BOC1* allele in common wild rice was found to reduce callus browning in *indica* rice transformation through decreasing cell senescence and death caused by oxidative stress [17]. OsSRO1a, another rice SRO member, acts as a negative regulator of JA signaling by acting as a mediator between OsMYC2 and the OsNINJA1-OsJAZ complex [18]. A wheat SRO protein Ta-sro1, which has PARP activity and DNA binding ability, was found to take part in seedling vigor and tolerance to salt stress through regulating redox homeostasis and maintaining genomic stability [9]. Transgenic *Arabidopsis* plants overexpressing *SRO* genes from several plant species, such as *MdRCD1* from apple (*Malus domestica*) [19], *ZmSRO1b* from maize (*Zea mays*) [20], *IcSRO1* from *Ipomoea cairica* [4], showed increased tolerance to various abiotic stresses.

In recent years, a total of 5, 6, 6, 6, 12, and 30 *SRO* family genes have been identified in rice [6,21], apple [19], maize [22], banana [23], Chinese cabbage [24], wheat [25], and tomato [26], respectively. However, no relevant systematic study about the expression and possible roles of *SRO* genes in sesame, which has high nutritional quality, but its production is severely threatened by adverse environmental stresses [27,28]. In the current study, four putative sesame *SRO* family genes were identified and performed a comprehensive analysis of them, including gene structures, protein conserved motifs, promoter *cis*-acting elements, tissue-, stress-, and phytohormone-related expression profiles. In addition, the overexpression of *SiSRO2b* in yeast enhanced tolerance to multiple abiotic stresses, including salt, osmotic, and oxidative stresses. Our findings lay the foundation for further functional characterization of *SiSRO* genes in plant stress tolerance and provide suitable candidate genes for future molecular breeding.

## 2. Results

### 2.1. Identification and Analysis of SRO Family Genes in Sesame

A total of four *SRO* genes were identified in sesame genome, and they were named as *SiSRO1a*, *SiSRO1b*, *SiSRO2a*, and *SiSRO2b* according to their evolutionary relationships (see below) and the nomenclature described by Jaspers et al. [3], respectively. Their characteristics including chromosomal position, corresponding protein length, MW, and pI were listed in Table 1. Three *SiSROs* (*SiSRO1a*, *SiSRO1b*, *SiSRO2a*) are distributed on two sesame linkage groups (LGs), LG2 and LG3 (Figure S1), while *SiSRO2b* is mapped to the unplaced scaffold (NW_011628063.1). Based on the result of MCScanX, no segmental duplication or tandem duplication events have been found among the *SiSRO* gene family. Physicochemical properties analysis revealed that the length of the *SiSRO* family ranged from 372 (SiSRO2b) to 560 (SiSRO1a) amino acids, the molecular weight ranged from 41.13 (SiSRO2b) to 62.73 (SiSRO1a), the pI ranged from 6.36 (SiSRO1a) to 8.29 (SiSRO1b). Subcellular localization analysis showed that SiSRO1a were distributed in the chloroplast, and the rest of the SiSROs were located in the nucleus.

### 2.2. Phylogenetic Analysis of SiSROs

**Figure 1:**
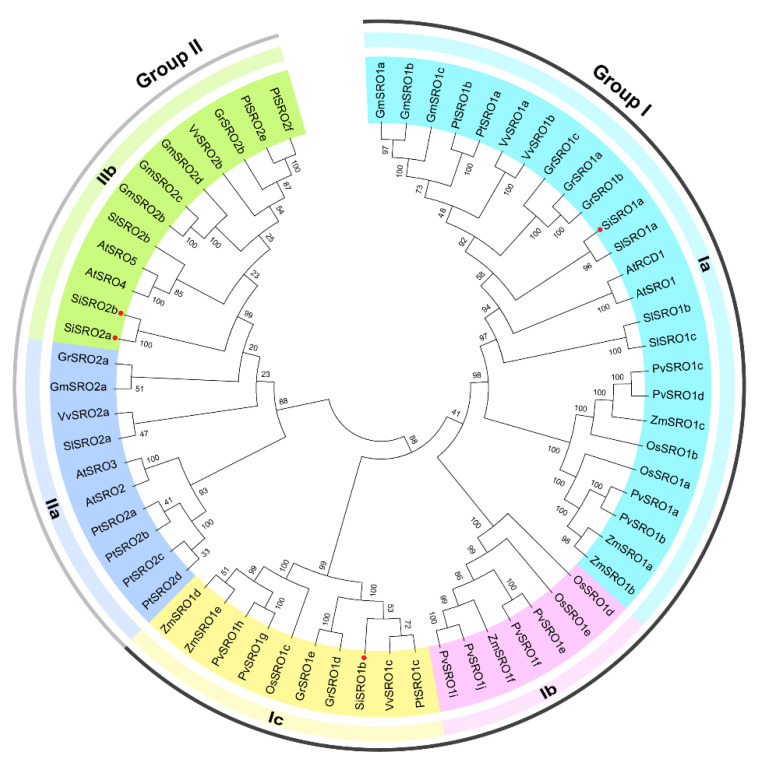
Phylogenetic analysis of SRO proteins from sesame and other plant species. The amino acid sequences of 64 SROs from sesame and other plant species were used to construct the phylogenetic tree based on the NJ method with 1000 bootstrap replicates in MEGA 5.0. The red dots represent SROs in sesame. The detailed information of SROs from representative plant species used in phylogenetic tree constructed were listed in Table S1.

To investigate the evolutionary relationships of *SRO* genes among sesame and other plant species, a phylogenetic tree was constructed based on 64 putative nonredundant SRO protein sequences from ten species, including Arabidopsis thaliana, *Glycine max*, *Oryza sativa*, Zea mays, *Gossypium raimondii*, Solanum lycopersicum, *Populus trichocarpa*, *Panicum virgatum*, *Vitis vinifera*, and *Sesamum indicum*. As a result, these SROs were divided into two groups, namely Group I and II (Figure 1). Group I contained both eudicot and monocot plants, and Group II contained only eudicots. Group I and II can be further subdivided into three and two subgroups, including Ia to Ic and IIa to IIb, respectively (Figure 1). Group Ia contains AtRCD1 and AtSRO1 as well as their homologs from all selected species, while group Ib members only from monocot plants. SiSRO1a and SiSRO1b were clustered into subgroup Ia and Ic, respectively, whereas SiSRO2a and SiSRO2b were clustered into subgroup IIb (Figure 1).

### 2.3. Intron-Exon Distribution and Motif Composition Analyses of SiSROs

**Figure 2:**
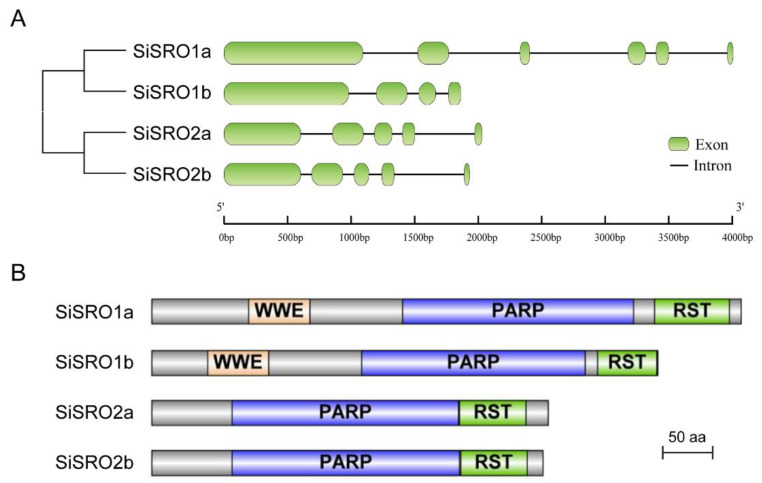
The exon-intron structure and conserved domain organization of SRO family in sesame. (**A**) Exon-intron structures of *SiSRO* genes. Exons and introns are shown as green rounded rectangles and black lines, respectively. (**B**) Schematic representation of conserved domains of SiSROs.

**Figure 3:**
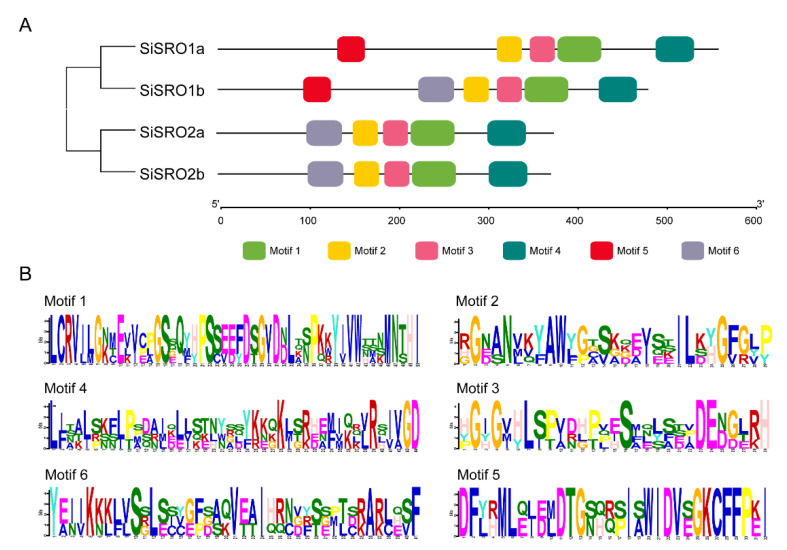
The conserved motif distributions of SRO family in sesame. (**A**). Distribution of the conserved motifs in SiSROs analyzed by MEME. Six motifs are marked by different colors. (**B**) Logo chart of the conserved motifs identified in SiSROs.

Structural diversity analysis of *SiSRO* genes showed that *SiSRO1a* had five introns, *SiSRO1b* contained three introns, whereas *SiSRO2a* and *SiSRO2b* each harbored four introns (Figure 2A). Domain analysis revealed that the PARP and RST domains are common to SiSRO proteins, and Group I SiSROs (SiSRO1a and SiSRO1b) additional contained one WWE domain at the *N*-terminal regions (Figure 2B and Figure S2). MEME was used to further determine the motif composition in SiSRO proteins, six conserved motifs were identified (Figure 3A) and the details of motifs were shown in Figure 3B. Amongst them, motifs 1–4 were appeared in all SiSROs, harboring PARP and RST domains, while motif 5 was specific to SiSRO1s (Figure 3A). In addition, SiSROs in the same subclade shared similar conserved motif management.

### 2.4. Cis-Acting Elements in the Promoters of SiSRO Genes

**Figure 4:**
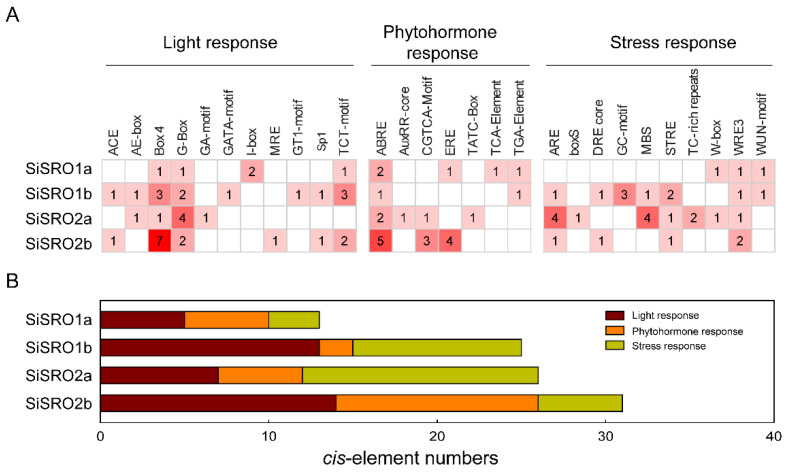
Predicted *cis* elements in the promoter regions of *SiSRO* genes. (**A**) Number of each *cis* element in the promoter region of *SiSRO* genes. Based on the functional annotation, the *cis* elements were classified into three major categories: light responsiveness-, phytohormone-, and stress-related *cis*-acting elements. The *cis* elements and their descriptions are listed in Table S2. (**B**) Sum of the *cis* elements in each gene and category.

The *cis*-acting elements in promoters of *SiSRO* genes were predicted by the online PlantCARE database. As shown in Figure 4A, a total of 95 *cis*-acting elements detected in the sequence of 1500 bp upstream of *SiSROs* transcription start position, including 39 light response elements, 24 hormone responsive elements, and 32 stress responsive elements. The most frequent elements were G-Box and Box4 involved in light responsiveness, ABRE (ABA responsive element), and ARE (essential for the anaerobic induction). There were significant differences in the types and abundance of elements among the four *SiSROs* promoters (Figure 4B). Element number was from 13 (*SiSRO1a*) to 31 (*SiSRO2b*), and type number was from 11 (*SiSRO1a*) to 17 (*SiSRO1b*). The promoter of *SiSRO2a* contained the most stress responsive elements (a total of 14), such as ARE, MBS, TC-rich repeats, boxS, W-box and WRE3. A total of 12 hormone responsive elements detected in the *SiSRO2b* promoter, including five ABRE, four ERE (ethylene responsive element) and three CGTCA-motif (MeJA-responsive element). The names and functions of each *cis*-acting element were shown in Table S2.

### 2.5. Expression Patterns of SiSROs in Various Tissues

**Figure 5:**
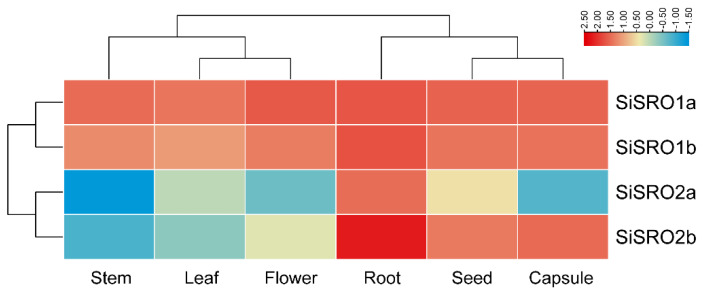
Transcription analysis of the *SiSRO* genes in different tissues. Log10-transformed RPKM values of each gene were used to construct heatmap. The expression level was shown in color as the scale.

To investigate the possible roles of *SiSROs*, their expression levels were analyzed in different sesame tissues, including leaf, root, stem, flower, seed, and capsule. As shown in Figure 5, *SiSRO1a* and *SiSRO1b* showed a similar expression pattern, and they had strong transcripts in all detected tissues. *SiSRO2b* displayed more abundantly expression in root, seed, and capsule than other tissues, with the lowest transcript levels in stem. However, *SiSRO2a* exhibited relatively lower transcript levels than other *SiSRO* genes, with the highest and lowest expression in root and stem, respectively (Figure 5).

### 2.6. Expression Profiles of SiSROs under Abiotic Stress Treatments

**Figure 6:**
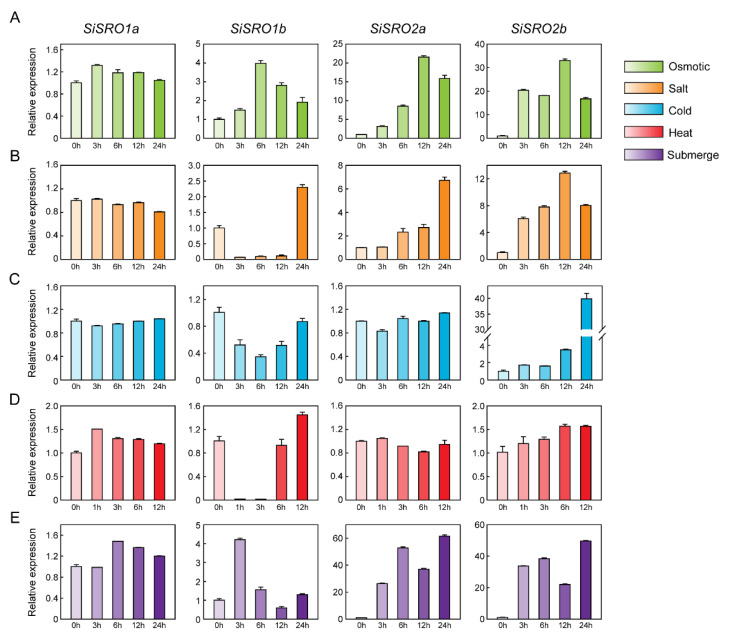
Expression profiles of the *SiSRO* genes in response to various abiotic stresses. (**A–E**) Expression of *SiSRO* genes under (**A**) osmotic (15% PEG 6000), (**B**) salt (150 mM NaCl), (**C**) cold (4C), (**D**) heat (42C), and (**E**) submergence treatments. Two-week-old seedlings were subjected to various stresses treatments. Relative expression levels of *SiSRO* genes were analyzed by qRT-PCR, using sesame *SiH3.3* gene as the internal control. Error bars indicate standard deviations (SD) based on three replicates.

Given the critical role of *SRO* genes in regulating abiotic stress response, qRT-PCR was used to determine the expression patterns of *SiSRO* genes under different abiotic stresses, including osmotic, salt, cold, heat, and submerge. Under osmotic stress, all four *SiSRO* genes exhibited sharply increased expression (fold change > 2) at different time points except *SiSRO1a* (Figure 6A). Under salt stress, the expression levels of *SiSRO2a* and *SiSRO2b* gradually increased and peaked at 24 h and 12 h, respectively (Figure 6B). *SiSRO1b* showed significantly decreased expression until 12 h and finally increased at 24 h, while *SiSRO1a* displayed slightly down-regulated expression at 24 h (Figure 6B). Under cold stress, the transcription of *SiSRO2b* was gradually increased and reached the highest at 24 h, while *SiSRO1b* was downregulated at all-time points (Figure 6C). The transcript abundance of *SiSRO1a* and *SiSRO2a* showed no obvious change. Under heat stress, the expression of *SiSRO1a* and *SiSRO2b* was slightly induced at all-time points, while *SiSRO1b* and *SiSRO2a* were downregulated at certain time points (Figure 6D). In addition, the transcription of *SiSRO1b* was markedly decreased at 1 h and 3 h, but its expression was sharply up-regulated thereafter at 6 h and peaked at 12 h. Under submerge stress, the expression of *SiSRO1b*, *SiSRO2a*, and *SiSRO2b* was significantly upregulated, and reached the highest level at 3 h, 24 h, and 24 h, respectively (Figure 6E). Overall, *SiSRO2b* was the most stress-responsive member of the SRO gene family in sesame, and strongly induced under osmotic, salt, cold, and submerge treatments. *SiSRO2a* was also upregulated under osmotic, salt, and submerge stress. The expression change of *SiSRO1a* under various abiotic stresses was smaller than that of other *SiSROs*.

### 2.7. Expression Profiles of SiSROs under Phytohormone Treatments

**Figure 7:**
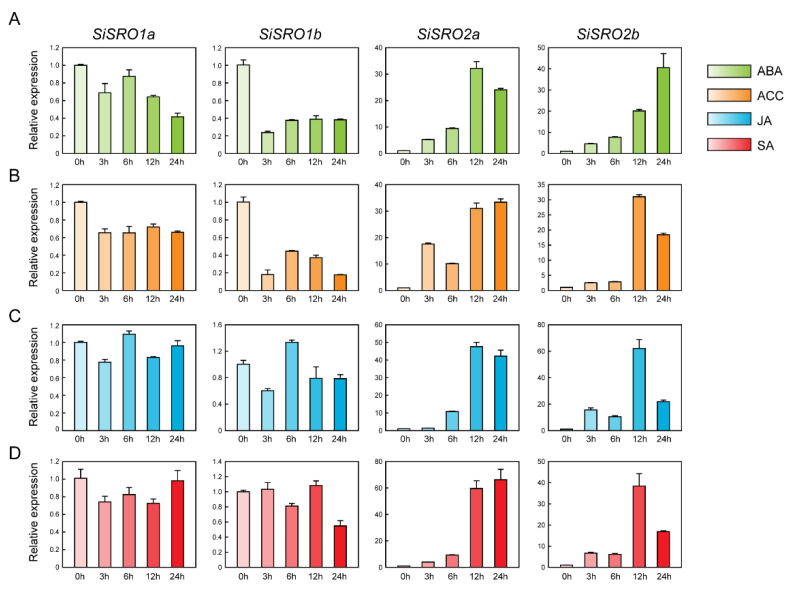
Expression profiles of the *SiSRO* genes in response to various phytohormone treatments. (**A–D**) Expression of *SiSRO* genes responses to (**A**) 100 μM abscisic acid (ABA), (**B**) 100 μM 1-aminocyclopropane-1-carboxylic acid (ACC), (**C**) 100 μM jasmonic acid (JA), and (**D**) 100 μM salicylic acid (SA). Two-week-old seedlings were treated with different phytohormone treatments. Relative expression levels of *SiSRO* genes were analyzed by qRT-PCR, using sesame *SiH3.3* gene as the internal control. Error bars indicate standard deviations (SD) based on three replicates.

We also examined the expression of *SiSRO* genes under the treatments of various phytohormones, including ABA, ACC, JA, and SA. Under ABA treatment, the expression of *SiSRO2a* and *SiSRO2b* displayed a gradually upregulated pattern and reached the highest level at 12 h and 24 h, respectively, while the expression of *SiSRO1a* and *SiSRO1b* was significantly decreased (Figure 7A). Similar expression patterns were also found under ACC treatment (Figure 7B). Under JA treatment, the expression of *SiSRO2a* and *SiSRO2b* showed gradual upregulation and reached the highest transcriptions at 12 h, while the expression of *SiSRO1b* was downregulated at the earlier time point (3 h), subsequently increased and peaked at 6 h, and finally declined at the later time points (12 h and 24 h) (Figure 7C). Like JA treatment, *SiSRO2a* and *SiSRO2b* showed remarkably upregulated transcript abundance during all SA experiment time points, and their expression peaked at 24 h and 12 h, respectively (Figure 7D). On the contrary, the expression of *SiSRO1a* and *SiSRO1b* was significantly decreased at certain time points under SA treatment (Figure 7D). Overall, the expression level of *SiSRO2a* and *SiSRO2b* was increased under all four phytohormones treatments, while *SiSRO1a* and *SiSRO1b* showed downregulation or no obvious change.

### 2.8. Protein-Protein Interaction Network of SiSROs 

**Figure 8:**
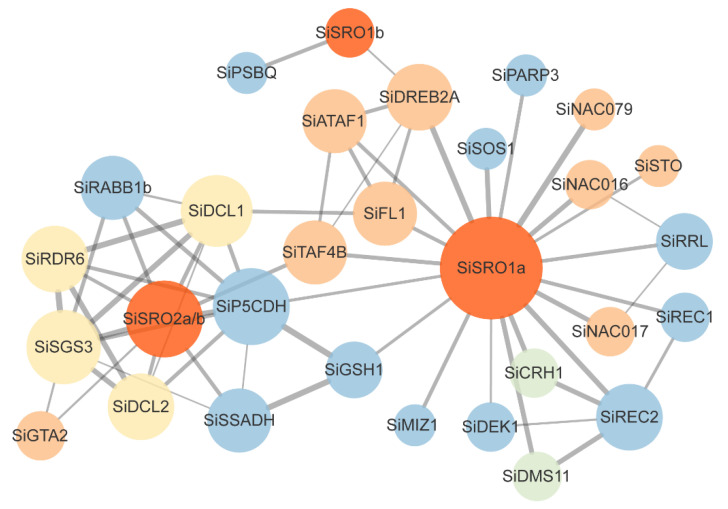
Protein interaction network analyses of SiSROs. Interaction networks were constructed based on homology of SiSROs in *Erythranthe guttata* using STRING database (https://string-db.org/, accessed on 11 October 2021). The sesame homologs of the SROs-interaction proteins in *E. guttata* were identified with reciprocal best BLASTP analysis and shown in network diagrams. Size of node indicates its connectivity degree, and the thickness/redness of the line represents the interaction score. Different colors represented different functional categories. The gene annotation of interaction proteins is available in Table S4.

To better understand the putative function and interactive relationship of SiSROs, a possible interaction network of sesame SROs based on their homologs from *Erythranthe guttata* (syn. *Mimulus guttatus*) (closely related species of sesame) was constructed in the STRING online database. The network constructed with medium confidence contains three SROs and 50 other interactive proteins from *E. guttata* (Table S3). Then, the homologs of these proteins were identified in sesame with reciprocal best BLASTP analysis and 42 putative SiSRO-interacting proteins were identified (Table S4). The interaction proteins with functional annotation were selected and interaction network of SiSROs was presented in Figure 8. Protein annotation showed the interaction proteins were mainly related to environmental stress responses, including the SOS1 functions in the removal of intracellular sodium and essential for salt tolerance, SSADH that are essential for maintaining the dynamic balance of ROS production under environmental stress, P5CDH involved in proline metabolism, and GSH1 that catalyzes the rate-limiting step of glutathione biosynthesis and regulates stress tolerance. SiSROs might interact with some TFs, including DREB2A, ATAF1, NAC016 and NAC017 that involved in abiotic stress responses, APETALA2 (AP2) that function in development of floral organ, STO that plays a role in light signal transduction, as well as GTA2 and TAF4B that regulate the transcription process. There were also some proteins participated in the synthesis of the small RNA species (such as DCL1, DCL2, SGS3 and RDR6) and epigenetic regulation (such as CHR1 and DMS11) in the interaction network diagram.

### 2.9. Co-Expression Network of SiSROs under Abiotic Stress Conditions

**Figure 9:**
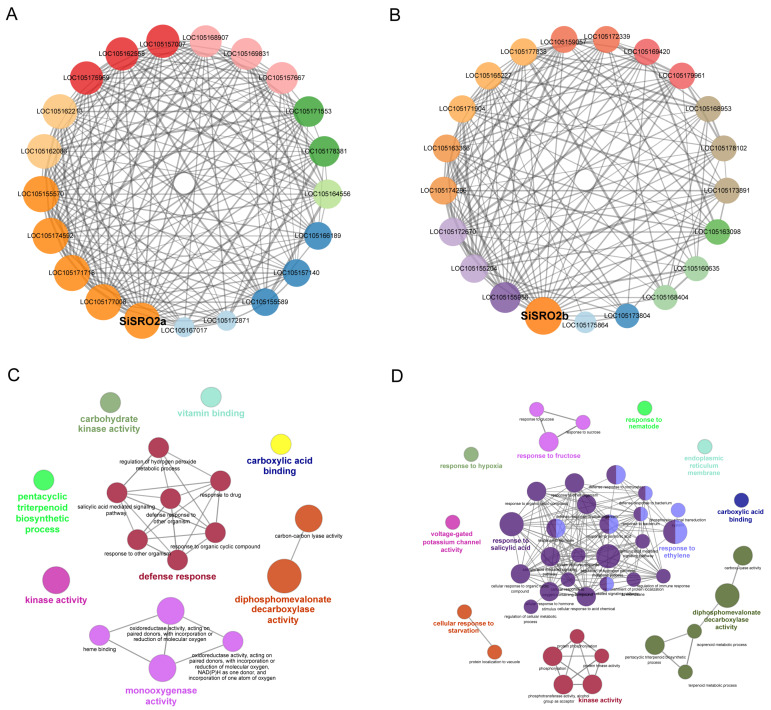
Co-expression analysis of *SiSROs* under abiotic stress. The co-expression network of *SiSRO2a* (**A**) and *SiSRO2b* (**B**). The co-expression neighbourhood of *SiSROs* was analyzed by mutual rank (MR)-based co-expression analysis based on transcriptomic data of sesame under various abiotic stresses. The 20 highly co-expressed genes with the smallest MR value are shown. GO enrichment of the co-expressed genes of *SiSRO2a* (**C**) and *SiSRO2b* (**D**). Functionally grouped network was generated in ClueGO with GO terms as nodes linked based on their kappa score level (≥0.3). Colors reflect the label of the most significant term per group. The node size represents the term enrichment significance.

We further conducted the co-expression network of two stress-responsive SiSROs, SiSRO2a, and SiSRO2b, using transcriptomic data of sesame under various abiotic stresses by Mutual Rank (MR)-based co-expression analysis. A total of 354 and 418 co-expressed genes (MR < 500) were found to be associated with *SiSRO2a* and *SiSRO2b* (Table S5)*,* respectively. The co-expression network of each *SiSRO* and its top 20 most correlated genes with the smallest MR value are displayed in Figure 9A,B. The genes highly co-expressed with *SiSRO2a* encoding E3 ubiquitin-protein ligase RMA1H1-like (LOC105177008), ethylene-responsive transcription factor ERF096 (LOC105171718), floral homeotic protein AGAMOUS-like (LOC105155570), pyruvate decarboxylase 1 (LOC105174592), two-component response regulator ARR5-like (LOC105157140), calcium-binding protein CML41 (LOC105166189) and so on. The highly co-expressed genes of *SiSRO2b* included expansin-like B1 (LOC105155956), BI1-like protein (LOC105163366), zinc finger protein AZF2-like (LOC105172670), ethylene-responsive transcription factor ERF071 (LOC105163098), and CASP-like protein PIMP1 (LOC105155204), MACPF domain-containing protein CAD1 (LOC105179961), and protein PHLOEM PROTEIN 2-LIKE A9 (LOC105175864). Gene ontology (GO) enrichment analysis revealed that defense response, regulation of hydrogen peroxide metabolic process, and pentacyclic triterpenoid biosynthetic process were major biological processes in which co-expressed genes of *SiSRO2a* were involved (Figure 9C). *SiSRO2b* and its co-expressed genes were enriched in response to salicylic acid, ethylene, and hypoxia, cellular response to starvation, pentacyclic triterpenoid biosynthetic process, and in response to fructose (Figure 9D).

### 2.10. Expression of SiSRO2b in Yeast Enhances Tolerance to Multiple Abiotic Stresses

**Figure 10:**
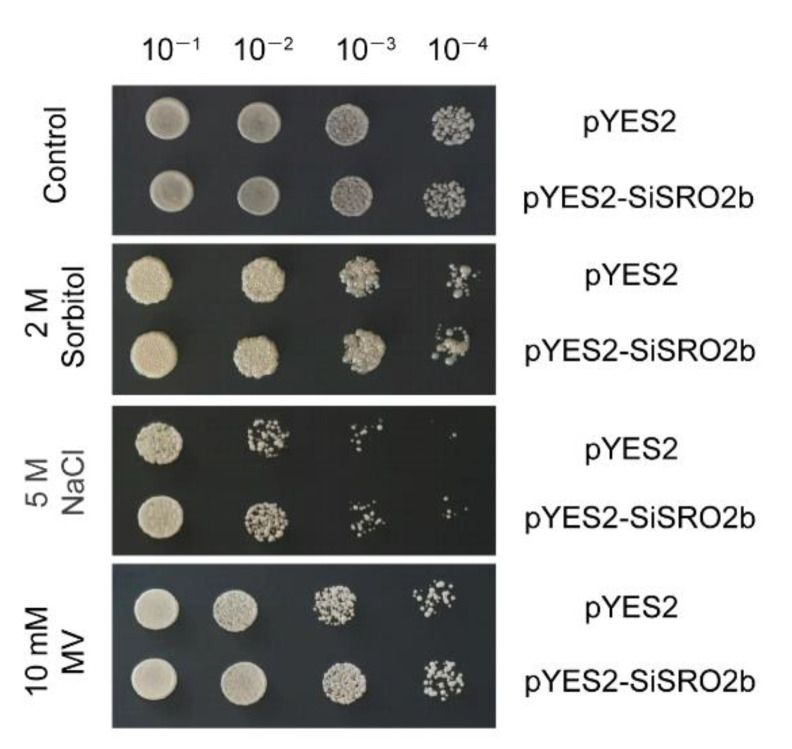
Effect of *SiSRO2b* expression in yeast under different stress treatments. Yeast cells harboring pYES2-SiSRO2b or the empty pYES2 were treated with 2 M sorbitol, 5 M NaCl or 10 mM MV for 24 h. The cells were then serially diluted and spotted on SD-Ura solid media for 2 days.

A yeast expression system was employed to investigate the role of SiSRO2b under various abiotic stresses. The growth of yeast cells transformed with pYES2-SiSRO2b plasmids or empty pYES2 (as a control) were surveyed after treated with sorbitol, NaCl or MV (methylviologen). As shown in Figure 10, no obvious difference was observed between the growth of yeast carrying empty pYES2 and under normal condition. However, yeast cells transformed with pYES2-SiSRO2b exhibited dramatically better growth than the control cells (harboring empty pYES2) after treated with sorbitol, NaCl or MV, suggesting that SiSRO2b confers osmotic, salt, and oxidative tolerance to transgenic yeast cells.

## 3. Discussion

To cope with various adverse conditions, plants have developed numerous physiological and metabolic response mechanisms by regulating a great many of stress-responsive regulatory and structural genes, so to survive in complex and diverse environments. As a small protein family unique to plants, SROs have been considered as key regulators which take part in a variety of abiotic stress and oxidative stress responses in plant. Here, we performed a comprehensive analysis of the SRO family in sesame, including genomic organization, tissue expression pattern, and gene expression profile in response to multiple abiotic stresses and phytohormone treatment. Four *SRO* genes were identified in sesame and were divided into two groups (Group I and II) according to phylogenetic analysis. Within the same group, SiSROs shared similar conserved domains and motif composition. SiSRO1a and SiSRO2b contained the WWE domain at the *N*-terminal, and clustered in Group I. Group II SiSROs, SiSRO2a, and SiSRO2b, only have the PARP domain and RST domain, lacking the WWE domain. Gene duplication events of the SRO family have been reported in tomato and wheat [25,26], but not detected in SiSROs.

Tissue atlases of gene expression provide useful information for elucidating the role of the genes in tissue development and function. *Arabidopsis RCD1* and *SRO1* are ubiquitously expressed and particularly highest in young developing tissues [7,13]. In rice, *OsSRO1d* and *OsSRO1e* showed constitutive expression in various tissues, while the expression of *OsSRO1c* in developing endosperm is much higher than other tissues [6]. The tissue-specific and ubiquitously expressed of SROs were also identified in tomato [26]. The results of the transcriptome data in this study showed that Group I SiSROs showed ubiquitously expressed in all detected tissues, suggesting they may function in the development of various tissues in sesame. Group II SiSROs were relatively highly expressed in root tissues. A vast number of studies have revealed that the *SRO* family genes can respond to abiotic stresses and stress-related hormones and play a role in plant abiotic stresses tolerance and hormone signaling pathways. For example, maize *ZmSRO1e* was significantly induced by multiple stress and function as negative regulator of anthocyanin synthesis to protect plants from excessive accumulated anthocyanins under abiotic stress [29]. *IcSRO1*, a homologous gene of *AtRCD1* in *Ipomoea cairica*, was upregulated by abiotic stress and ABA treatment. Over-expression of *IcSRO1* conferred salt and drought tolerance in transgenic *Arabidopsis* [4]. In this study, the *cis* elements analysis of *SiSRO* genes suggested that *SiSROs* are widely involved in response to abiotic stresses and phytohormone treatments, which is consistent with the results reported in maize, wheat, tomato, and banana [23,25,26,29]. Subsequently, the expression levels of *SiSRO* genes under various abiotic stresses (osmotic, salt, cold, heat, and submerge) and different hormone (ABA, ACC, JA and SA) treatments were analyzed by qRT-PCR. Almost all *SiSROs* were found to be induced or repressed by one or more exogenous stimulus (Figure 7 and Figure 8), but the expression patterns of the various *SROs* are different. The SiSROs members in Group II exhibited more responsive to stress than those in Group I, which is consistent with more stress and hormone related *cis* elements harbored in their promoters (Figure 6). *OsSRO1c* and *ZmSRO1e* were the most stress responsive *SRO* genes in rice and maize, respectively, and their important roles in abiotic and oxidative stress have been conformed [8,29]. It is worth noting that *SiSRO2b* was the most stress responsive of the sesame *SRO* family, implying its key role in abiotic stress response.

SRO proteins are considered as cellular hub proteins since they interact with large numbers of proteins, predominantly transcription factors. At least 21 TFs belonging to different TF family, including NAC, AP2/ERF, and bHLH, are interacted with AtRCD1 and function in various developmental and stress response signaling pathways [7]. Rice SRO protein OsSRO1 was also found interacted with 29 *Arabidopsis* transcription factors by large-scale pair-wise interaction screening against an *Arabidopsis* transcription factor library [6]. Several studies indicated that AtRCD1 negatively regulates its interaction partners, and interactions of AtRCD1 with TFs are essential for regulation of various biological processes, including leaf senescence, stress responses, and organellar (chloroplast and mitochondrial) ROS signal processing [5,30]. In the present study, we performed protein-protein interaction network analysis using the STRING database and identified 42 putative SiSROs-interacting proteins. Some of these interaction proteins, such as DREB2B, NAC017, STO, and SOS1, are known interaction partners of AtRCD1. Functional annotation analysis revealed that many SiSROs-interacting proteins are involved in abiotic stress response. For example, the *NAC016* homologous in *Arabidopsis* is involved in drought stress response by downregulate *AREB1* expression through a trifurcate feed-forward regulatory model involving NAP [31]. *GSH1* encoding gammaglutamyl cysteine synthase, the rate-limiting enzyme of GSH biosynthesis, was important for plant performance in response to different abiotic stresses [32]. Gene co-expression networks have been widely used to predict protein function by linking unknown functional proteins to known biological processes through their associated genes [33,34]. Genes co-expressed with stress-responsive sesame SROs, *SiSRO2a* and *SiSRO2b*, were identified based *on* transcriptomic data of sesame exposed to various abiotic stresses. GO enrichment analysis showed that *SiSRO2a* perform its function by regulating defense response, hydrogen peroxide metabolic process, while *SiSRO2b* associated genes were mainly participated in biological processes related to hormonal and stress responses. Although the function of these co-expressed genes in sesame is largely unknown, their homologues from other plant species have been linked to various stress response pathways. LOC105177008 is the most strongly co-expressed gene (MR value = 2) of *SiSRO2a*, which encoding an E3 ubiquitin-protein ligase. Rma1H1, the homologue of LOC105177008 in hot pepper (*Capsicum annuum*) and conferred highly tolerance to drought stress in transgenic *Arabidopsis* by regulating aquaporin levels via ubiquitination [30]. *LOC105171718* is homologue to *Arabidopsis ERF96* and highly co-expressed (MR value = 2.45) with *SiSRO2a*. Wang et al. found that overexpression of ERF96 activated expression of some NaCl-responsive genes and enhanced salt tolerance in *Arabidopsis* [35]. For *SiSRO2b*, the most strongly co-expression gene *LOC105155956* (MR value = 2.83) encoding expansin-like B1. *Brassica rapa* expansin-like B1 *(BrEXLB1)* was reported positively associated with drought tolerance of *B. rapa* plants [36]. Homologue gene of *Arabidopsis* zinc finger protein AZF2 was found co-expressed with *SiSRO2b* (MR value = 3.87), and AZF2 was reported acts as a transcription repressor to inhibit plant growth under adverse environment [35]. Other co-expressed gene of *SiSRO2b*, *LOC105163098* encoding ethylene-responsive transcription factor, and its homologous in *Arabidopsis* (*AtERF71*) and soybean (*GmERF75*) were found mediates osmotic and hypoxia stress response [36,37]. Taken together, the interaction and co-expression gene network analysis suggested that *SiSRO* genes, especially *SiSRO2a* and *SiSRO2b*, are widely involved in stress response by regulating transcription factors and other proteins.

Yeast (*Saccharomyces cerevisiae*) is a widely used eukaryotic model for understanding basic molecular mechanisms and biological processes, which has been used as a tool for quick identification of genes involved in abiotic stress tolerance. RCD1 (also named as CEO1) was found to complement the oxidative stress sensitivity in yeast strain WYT, which was deficiency in the redox sensor YAP1 [11]. Overexpression of RCD1 also improved the oxidative stress tolerant in the wild-type yeast [11]. Sharma et al. found that co-transformation of rice SRO protein OsSRO1a and its interaction pattern OsRBD1 in yeast enhanced tolerance to salinity, osmotic, as well as oxidative stress induced by methylglyoxal [21]. Many other types of plant genes have also demonstrated their role in abiotic stress tolerance through the yeast expression system. For example, overexpression of the translation initiation factor eIF1A (BveIF1A) from halotolerant plant sugar beet improved salt tolerance of sodium-sensitive yeast strain [31]. Alavilli et al. reported that overexpression of *HvPIP2;5*, a barley aquaporin gene, enhanced tolerance to salt and osmotic stresses in yeast [32]. In this study, the yeast expression system was used to characterize the function of *SiSRO2b* in stress tolerance. The results showed that the yeast cells harbored *SiSRO2b* showed greater tolerance to osmotic, salinity, and oxidative stresses, indicating *SiSRO2b* is involved in tolerance to a variety of abiotic stresses.

## 4. Materials and Methods

### 4.1. Identification of SRO Family Genes and Phylogenetic Analysis

To screen the SRO proteins in sesame (*Sesamum indicum* L.), the Hidden Markov Model (HMM) profile of WWE domain (PF02825), PARP domain (PF00644), and RST domain (PF12174) were download from the Pfam database [38] and used for local Hidden Markov Model (HMM) search against the whole-genome protein sequences of sesame (https://ftp.ncbi.nlm.nih.gov/genomes/refseq/plant/Sesamum_indicum/latest_assembly_versions/GCF_000512975.1_S_indicum_v1.0/, accessed on 15 April 2021) by HMMER3.0 [39]. A local BLASTP search was also performed against sesame protein sequences using known SROs from *Arabidopsis* [3] and rice [6] as queries with a cut-off e-value of 1e-10. SRO proteins in *Glycine max*, Solanum lycopersicum, Zea mays, *Gossypium raimondii*, *Populus trichocarpa*, *Panicum virgatum*, and *Vitis vinifera* were identified by BLASTP searches in Phytozome v13 (https://phytozome-next.jgi.doe.gov/, accessed on 15 April 2021). Finally, all candidate protein sequences were further confirmed by SMART (http://smart.embl-heidelberg.de/, accessed on 15 April 2021) [40], and InterPro (http://www.ebi.ac.uk/interpro/, accessed on 15 April 2021) [41]. All identified SRO proteins were aligned using ClustalX (version 1.83) program [42]. Then, the unrooted phylogenetic tree was constructed in MEGA5 software [43] based on the neighbor-joining (NJ) method with 1000 bootstrap replicates. Molecular weight and isoelectric point of SiSRO proteins were analyzed by the Compute pI/Mw tool (http://web.expasy.org/compute_pi/, accessed on 17 April 2021). Subcellular localization of SiSROs was predicted by Plant-mPloc server (http://www.csbio.sjtu.edu.cn/bioinf/plant-multi/, accessed on 17 April 2021).

### 4.2. Gene Structure, Protein Conserved Motifs and cis-Acting Element Analysis

The gene structure of SiSROs was analyzed by TBtools software [44] based on gene-structure annotation file in GFF3 format of sesame. Conserved motifs of SiSROs were analyzed using MEME (Multiple Em for Motif Elicitation) v5.3.3 (http://meme-suite.org/tools/meme, accessed on 15 June 2021) [45], with the default parameters. The XML file storing motif pattern information obtained from MEME was used to generate schematic diagrams of motif distribution by TBtools software [44]. The 1500 bp sequences upstream of transcription start position from each *SiSRO* genes were extracted from genomes of sesame. The *cis*-acting regulatory elements were predicted by the online tool PlantCARE (http://bioinformatics.psb.ugent.be/webtools/plantcare/html/, accessed on 9 June 2021).

### 4.3. Protein Interaction and Co-Expression Analysis

The possible interaction network of sesame SROs was constructed based on their homology from *Erythranthe guttata* (closely related species of sesame) in the STRING online database (https://string-db.org/, accessed on 11 October 2021). Then, sesame homologs of SROs interaction proteins in *Erythranthe guttata* were identified by reciprocal best BLASTP analysis. Afterthat, interaction network of SiSROs was constructed in Cytoscape 3.7.1.

Transcriptomic data of sesame under various abiotic stresses, including drought [46,47], salt [48] and waterlogging [49], was used for identification of co-expressed genes of *SiSROs* under abiotic stress conditions. The co-expression neighbourhood of *SiSROs* was analyzed in MutRank software using Mutual Rank (MR)-based co-expression analysis [50], and the top 20 highly co-expressed genes (with the smallest MR value) were selected to construct co-expression network in Cytoscape 3.7.1. Enrichment analyses of Gene Ontology (GO) of SiSROs co-expression genes (MR < 500) were performed using the ClueGO plugin in Cytoscape [51]. 

### 4.4. Plant Materials and Treatments

Sesame cultivar Zhongzhi No. 13 was grown hydroponically in a growth chamber (16 h light/8 h dark cycle at 28 °C). Two-week old seedlings were exposed to various abiotic stresses and phytohormone treatment under continuous light according to previous studies [6,52] with modifications. Namely, seedlings were transferred in culture solution containing either a hormone solution (100 μM ABA, SA, JA or ACC), 15% PEG6000, or 150 mM NaCl. Cold and heat stress were applied by transferred the seedlings to a growth chamber at 4 °C or 42 °C, respectively. For submergence stress, the seedlings were completely submerged in a plastic tank filled with water. Controls were kept on culture solution containing an equal volume of the corresponding solvent (ethanol or water). The shoots of seedlings with different treatments were harvested at 0, 3, 6, 12, and 24 h after treatment with three replicates. Only the shoots of seedlings under heat stress were sampled at 0, 1, 3, 6, and 12 h. Samples were immediately frozen in liquid nitrogen and stored in −80 °C for RNA extraction. 

### 4.5. RNA Isolation and Quantitative Real-Time RT-PCR

Total RNA was isolated with the EASY spin Plus kit (Aidlab, Beijing, China) according to the manufacturer’s instruction. The first strand cDNAs were synthesized by the HiScript II 1st Strand cDNA Synthesis kit (Vazyme Biotech, Nanjing, China). Quantitative real-time RT-PCR (qRT-PCR) was performed using Roche LightCycler 480 real-time PCR system as described by You et al. [53]. The relative expression levels were analyzed using 2^−ΔΔCT^ method [54]. The gene-specific primers are listed in Table S6.

### 4.6. Cloning and Functional Characterization of SiSRO2b in Yeast

The full-length cDNA sequence of *SiSRO2b* was isolated from drought-treated leaf sample of stress-resistant sesame variety ‘Zhongzhi 75′ by RT-PCR using gene-specific primers SRO2bFL listed in Table S6. The PCR fragment was inserted into the pYES2 vector (Invitrogen Life Technologies, Carlsbad, CA, USA) under control of *GAL1* promoter by homologous recombination using the ClonExpress II One Step Cloning Kit (Vazyme Biotech, Nanjing, China). The pYES2-Si*SRO2b* and empty control plasmids were transformed into INVSc1 yeast strain (Invitrogen Life Technologies, Carlsbad, CA, USA) by lithium acetate method [55]. The transformants were then selected by growth on SC-Ura medium with 2% (*w*/*v*) glucose at 30 °C. 

For the stress tolerance assay, yeast cells carrying pYES2 or pYES2-*SiSRO2b* were grown in SC-Ura liquid medium with 2% (*w*/*v*) glucose at 30 °C overnight. To induce the expression of *SiSRO2b* gene, the overnight cultures were adjusted to OD_600_ = 0.4 in induction medium (SC-Ura medium containing 2% *w*/*v* galactose) and cultivated at 30°C for 36 h. Then cultures were adjusted to an equal cell number and collected for stress treatments. Yeast cells were treated either with 5 M NaCl (salt stress), 2 M sorbitol (osmotic stress), or 10 mM MV (oxidative stress) for 24 h. Subsequently, serial dilutions were spotted onto SC-Ura agar plates and incubated at 30 °C. Each experiment was carried out in triplicate.

## 5. Conclusions

In current study, four *SRO* genes (*SiSRO1a*, *SiSRO1b*, *SiSRO2a*, and *SiSRO2b*) were identified in old oilcrop sesame (*Sesamum indicum* L.). Constitutively and tissue-specific expressed *SiSRO* genes were identified based on transcriptomic analysis. The results of qRT-PCR analysis indicated that *SiSROs*, especial *SiSRO2a* and *SiSRO2b* from group II, showed significantly responsive to various abiotic stresses and hormones. The critical regulatory roles were implied by co-expression and interaction analysis. Moreover, the function of SiSRO2b in stress tolerance was verified using yeast expression system. Overall, our work lays a foundation for further functional characterization of *SiSRO* gene family, as well as breeding improvement for stress resistance in sesame.

## Figures and Tables

**Table 1:** SRO gene family in sesame.

Gene Name	Gene ID	Linkage Group	Start (bp)	End (bp)	No. of Exon	Protein Length (aa)	MW (kD)	pI	Subcellular Localization
SiSRO1a	LOC105155511	LG2	6894030	6901957	6	560	62.73	6.36	Chloroplast
SiSRO1b	LOC105159067	LG3	21219139	21221593	4	481	54.39	8.29	Nucleus
SiSRO2a	LOC105157214	LG3	3993893	3996925	5	377	42.35	6.43	Nucleus
SiSRO2b	LOC105179407	Unplaced Scaffold NW_011628063.1	287017	290048	5	372	41.13	6.86	Nucleus

## Data Availability

Gene sequence information of SROs in sesame is available at the NCBI database (https://www.ncbi.nlm.nih.gov, accessed on 11 October 2021).

## Supplementary Materials

The following are available online at https://www.mdpi.com/article/10.3390/ijms222313048/s1.
