# Supplementary material for: Comprehensive Analysis of SRO Gene Family in Sesamum indicum (L.) Reveals Its Association with Abiotic Stress Responses
Source: Int J Mol Sci. 2021 Dec 2;22(23):13048. doi: 10.3390/ijms222313048 (PMC8657681; doi:10.3390/ijms222313048)
Supplement: Supplementary file 1 [file ijms-22-13048-s001.zip › Figures S1 and S2.pdf]

Comprehensive Analysis of *SRO* Gene Family in Sesame (*Sesamum indicum* L.) Reveals Its Association with Abiotic Stress Responses

Aili Liu [1], Mengyuan Wei [1], Yong Zhou [2], Donghua Li [1], Rong Zhou [1], Yanxin Zhang [1], Xiurong Zhang [1], Linhai Wang [1,*] and Jun You [1,*]

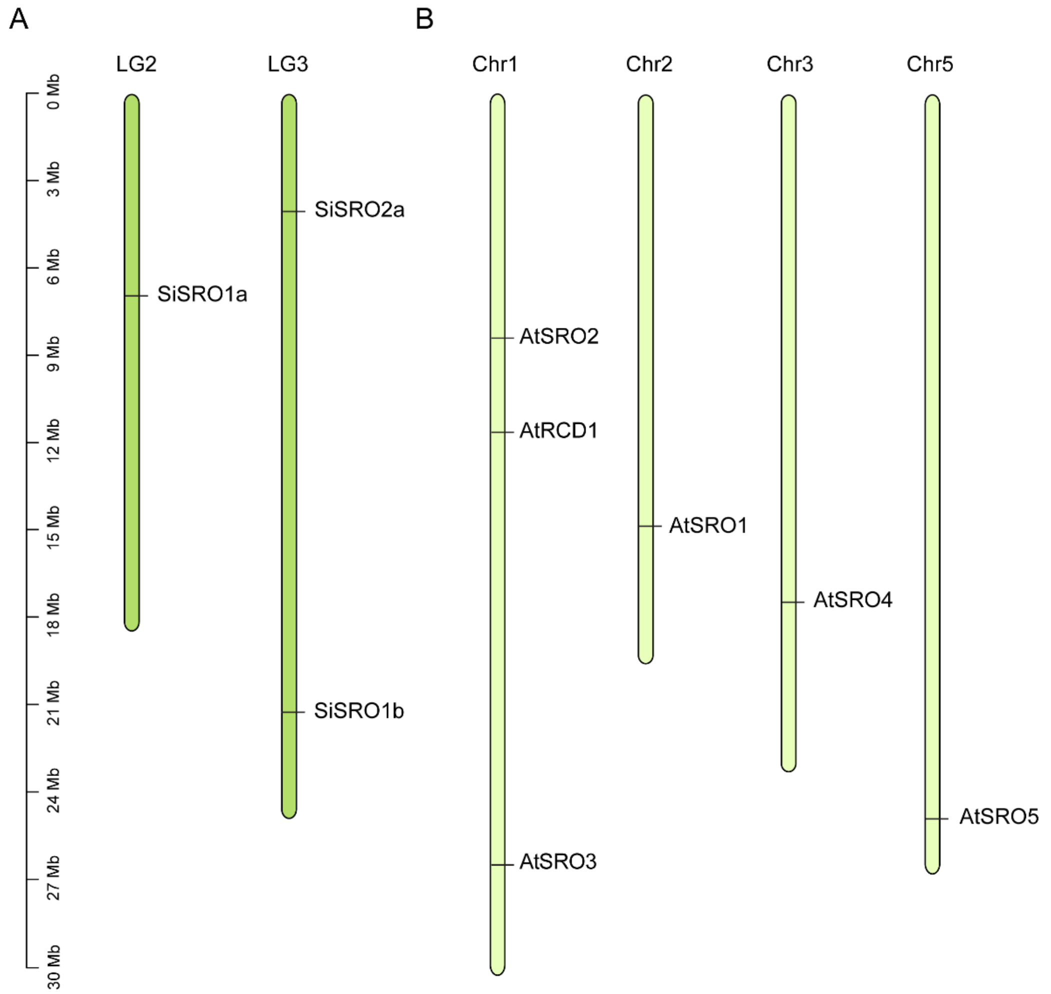

Figure S1. Distribution of *SRO* genes on sesame linkage groups (A) and *Arabidopsis* chromosomes (B).

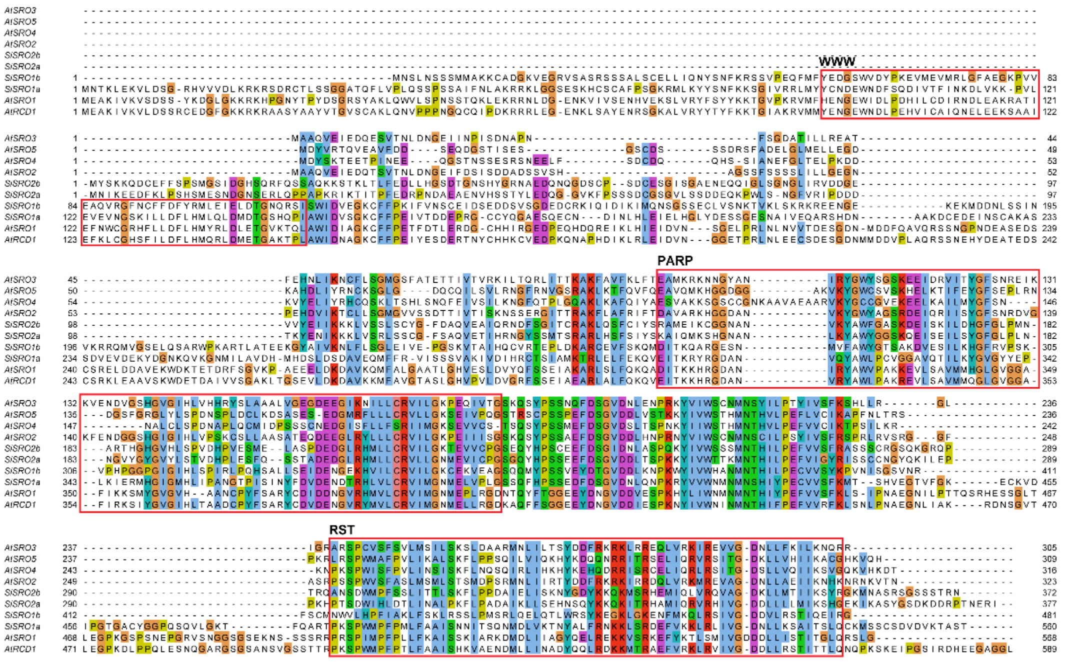

Figure S2. Multiple sequence alignment of SRO proteins from sesame and *Arabidopsis*.
